# Empowering medical students as agents of curricular change: a value-added approach to student engagement in medical education

**DOI:** 10.1007/s40037-019-00547-2

**Published:** 2019-12-10

**Authors:** Joseph R. Geraghty, Alexandria N. Young, Tiffani D. M. Berkel, Eric Wallbruch, Julie Mann, Yoon Soo Park, Laura E. Hirshfield, Abbas Hyderi

**Affiliations:** 1 Medical Scientist Training Program, University of Illinois at Chicago, Chicago, IL USA; 2 Department of Medical Education, University of Illinois at Chicago, Chicago, IL USA; 3 Department of Clinical Science, Kaiser Permanente School of Medicine, Pasadena, CA USA

**Keywords:** Medical students, Student engagement, Curriculum, Value-added medical education

## Abstract

One area in which medical students can add significant value is medical education, and involving them as key stakeholders in their education can have a profound impact on students and the institutions that serve them. However, detailed descriptions of the structure, implementation and quality of programs facilitating student engagement are lacking. We describe the structure of a novel student engagement program at the University of Illinois College of Medicine-Chicago (UICOM-Chicago) known as the Student Curricular Board (SCB). We surveyed 563 medical students across all levels of training at our institution in order to examine the impact of this program, including its strengths and potential areas of improvement. The SCB serves as a highly structured and collaborative student group that has far-reaching involvement from course-level program evaluation to longitudinal curriculum design. Medical students overwhelmingly valued opportunities to be involved in their curriculum. Students with the greatest exposure to the SCB were more aware of specific program initiatives and expressed increased interest in academic medicine as a career. By highlighting this innovative student engagement program, we aim to share best practices for a highly structured, value-added approach to medical student engagement in medical education that is applicable to other medical schools and student leaders.

## Introduction

There has been a recent push to shift our understandings about the way medical students can ‘add value’ to healthcare systems [1–3]. However, few scholars have investigated the potential for them to add value to their own medical curricula [4]. Further, while research has shown that student engagement in medical curricula is appreciated by administrators, less is known about how these opportunities are perceived by students themselves [5–11]. Student engagement programs vary widely and only rarely are these programs described thoroughly enough to allow adoption by other schools [8]. Furthermore, the majority of these established student groups focus on evaluation of pre-clinical courses [7, 9]. Detailed descriptions of the structure, process and benefits of student engagement programs may facilitate implementation and highlight their value. Many institutions involve students as stakeholders in curriculum development during large-scale curricular transformation via participation in administrative committees, focus groups and task forces; it is less common to see students fully integrated into ongoing curricular management and quality improvement processes such as those required for accreditation by the Liaison Committee on Medical Education (LCME) [12].

We developed an innovative program at the University of Illinois College of Medicine-Chicago (UICOM-Chicago) known as the Student Curricular Board (SCB) to offer medical students an opportunity to provide direct feedback about their pre-clerkship, clerkship and post-clerkship courses, as well as to contribute to larger-scale curricular discussions. Here, we (1) provide a description of the SCB to facilitate implementation elsewhere; and (2) examine student perceptions on these opportunities to add value to their own education. Findings are presented with implications for the health professions community in developing student engagement programs, as well as understanding their impact on students.

## The student curricular board

**Fig. 1:**
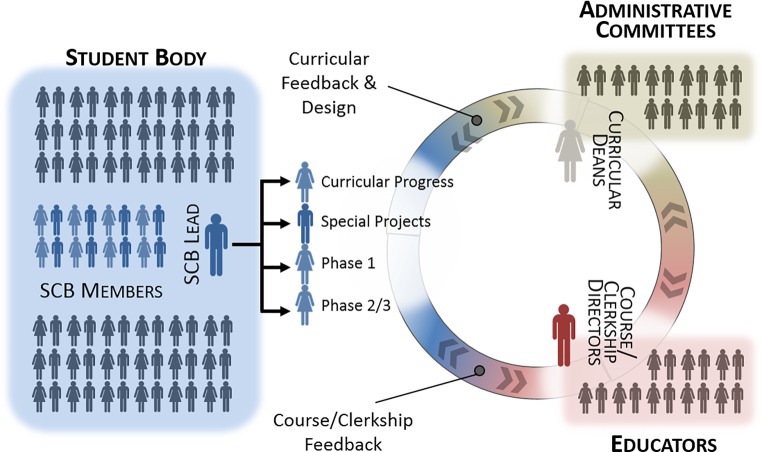
Strategic design of the Student Curricular Board (*SCB*) at the University of Illinois College of Medicine-Chicago (UICOM-Chicago) allows for iterative discussion and feedback loops between the student body, faculty and administration. The SCB consists of four branches: the *phase* *1* branch oversees program evaluation of pre-clerkship courses, the *phase* *2/3* branch oversees the program evaluation system in core clerkships and post-clerkship curriculum, the *curricular progress* branch employs a scholarly approach to collaborative decision-making on large-scale curriculum transformation and longitudinal curricular themes, and the *special projects* branch identifies tangible short-term projects that lead to curricular improvements

UICOM-Chicago’s history of collaboration between students and faculty has resulted in improvements to educational outcomes and student experiences [13]. In this tradition, the SCB was established in 2012 and originally focused on pre-clerkship course program evaluation. However, given high levels of student and faculty engagement, we expanded to increase student participation in curricular change throughout all years of training (Fig. 1).

The SCB operates under the medical student council and is financially supported by the Office of Curricular Affairs, uniting student and administrative resources. Student leaders appoint members via formal application and interview, recruiting roughly 20 student members per year (approximately 11% of each class), for a total of 80 across all class-years. Members are appointed as first-year students with the goal of a longitudinal experience throughout medical school. They are trained in conflict resolution, program evaluation and curricular design during a 2‑h orientation run by SCB members and expert medical education faculty, with workshops held throughout the year on additional topics (e.g. qualitative research methods). Once fully trained, members are assigned as voting members on administrative committees and to one of four SCB branches (Fig. 1); depending on assigned role and students’ enthusiasm, time commitment varies.

The phase 1 branch oversees program evaluation of pre-clerkship courses, in which a pair of student liaisons are assigned to each course, transmitting curated feedback in real time to instructors and relaying messages back to the student body. Through an iterative process, students and course directors review feedback from current and past student bodies, discuss potential improvements to the courses, and present these in formal reports to administrative committees. These reports are available to the student body and are reviewed by senior administration to make formal year-end recommendations on course structure and content. Similarly, the phase 2/3 branch oversees student-driven feedback efforts on the clerkship and post-clerkship curriculum. Student liaisons report anonymous feedback to clerkship directors in real time and more formally at the end of each rotation and in annual reviews. Through this process, student liaisons gain an opportunity to discover both the opportunities and challenges that faculty and administrators face when designing and evaluating preclinical and clinical curriculum.

The curricular progress and special projects branches have a macro-curricular focus that extends beyond single courses or rotations. The curricular progress branch directly incorporates students into decision-making during large-scale curriculum reform, such as a recent overhaul of the UICOM medical curriculum to a systems-based, pass/fail, integrated structure. Students make recommendations for feasible solutions to identified issues, employing a scholarly approach through literature review, environmental scanning, focus groups and data analysis. They focus on topics related to the LCME standards and the Association of American Medical College’s pilot project on Core Entrustable Professional Activities for Entering Residency—UICOM-Chicago is one of ten schools participating in the pilot—and collaborate with committees on diversity and wellness. The special projects branch applies similar methods and allows students to pitch smaller-scale, tangible projects that could lead to curricular improvements, such as collaborating with staff to better disseminate grades electronically and implementation of single curricular experiences.

Thus, the structure of the SCB was designed strategically, taking into account various ways in which students can add value to curriculum development and evaluation. More information can be found at https://cmsc.org.uic.edu/student-curricular-board/.

## Evaluation

We surveyed UICOM-Chicago students from January to April 2017 to examine perceptions about the impact of the SCB. All students were eligible to participate. Responses were voluntary and uncompensated. Survey questions were developed iteratively from literature review and discussion among members of the research group, three of whom were student leaders. Our survey included Likert-type questions and optional open-ended responses. The UIC Institutional Review Board approved this study and the evaluation conducted was carried out in accordance with the Declaration of Helsinki.

Responses to Likert-type questions were dichotomised based on whether the student agreed or not [14]. Chi-squared tests were used to examine differences across student responses. Cronbach’s alpha was used to examine the internal-consistency reliability of survey items and was found to be 0.91. Data analysis was conducted using Stata 14 (College Station, TX, USA). Two of the authors independently reviewed the open-ended data and performed a thematic analysis of all responses [15]. Exemplary quotations are provided that represent relevant themes and provide context for our quantitative findings [15].

## Results

Of 753 total students across all years, 563 (74.8%) responded. Of these, 63 students were formally involved in the SCB (11.2% of all respondents, 78.8% response rate amongst SCB members). Nearly all (96%) students agreed that opportunities for involvement in curriculum are important, and there was no difference between students involved with the SCB compared to those who were not (92% vs 93%, χ^2^ = 0.03, *p* = 0.862) nor between pre-clerkship and clerkship students (91% vs 94%, χ^2^ = 1.5791, *p* = 0.209). A majority (>50%) of students viewed the SCB and opportunities for student engagement favourably across the range of survey items, agreeing that administrators value student input (77%), that the SCB advocates for all students (80%) and fosters collaborative relationships amongst faculty and students (81%), and that changes to curriculum driven by the SCB have improved the student experience (71%). Notably, students involved in the SCB and senior students had more favourable views for these items and were more likely to be aware of specific curricular initiatives (*p* < 0.05 for all items).

We next categorised 1,257 comments into key themes (Tab. 1). Themes about strengths of student engagement included:

- *Empowerment and ownership*: Students feel a sense of ownership over their own education, resulting in greater trust and respect for faculty and administration.
- *Student voice and advocacy*: Students appreciate that their voice is heard and valued. Students serve as advocates, developing valuable skills important for future physicians.
- *Student-faculty collaboration*: The SCB encourages greater communication and collaboration between students, faculty and administration. Students appreciate the complexity of medical education, gain exposure to mentors, and feel they add value.
- *Organised student feedback*: The SCB allows for synthesis of ideas and greater advocacy for change, ensuring that the program evaluation process is efficient and curricular decisions are not based on the opinion of individual students.
- *Reactivity to specific issues*: Students appreciated peer responses to certain incidents, such as holding town hall meetings to address pressing issues directly and in real time.
- *Exposure to academic medicine*: Involved students reported increased interest in and better preparation for careers in academic medicine.

**Table 1:** Thematic analysis and representative quotations on the impact of student engagement in curriculum through the Student Curricular Board (*SCB*)

	Theme	Examples of SCB-led initiatives	Representative quotations
*Strengths of student engagement in curriculum*	*Empowerment and ownership*	Direct access to faculty/administrators, voting membership and standing agenda time in curricular committees, leadership experience, tangible curricular improvements, reports utilised in administrative decision-making	‘Rather than complaining about courses, I feel like I can actively improve them!’
			‘It has greatly improved the students’ experience. Oftentimes, curriculum decisions are made for students without student input. At UICOM, we are part of the decisions that affect us’
	*Student voice and advocacy*	Formal feedback systems (surveys, town hall meetings), representation on administrative committees, direct access to faculty/administrators, liaison responsibilities between students and course directors	‘Even though I am not very involved in the SCB, I feel they really advocate for improved experiences. There are some changes that are evident even this year compared to last year that I feel have created a better learning experience’
			‘I personally feel that student involvement in curriculum is essential and am fortunate to be at a school where the administration and faculty value student input’
	*Student-faculty collaboration*	Direct access to faculty/administrators, voting membership and standing agenda time in curricular committees, integration of the Office of Curricular Affairs, culture of valued partnership and inclusion on curricular teams	‘The ability to interface with upper-level faculty and administration is a really powerful concept. It gave me a sense that there wasn’t some large chasm between a lowly M1 student and the folks that are directing and adapting the curriculum’
			‘Important information has been efficiently passed back and forth between the instructors and student body, and I really feel like I have a say in my education’
	*Organised student feedback*	Formal feedback systems (surveys, town hall meetings), organisational framework with formal recruitment and training, integration with student government and the Office of Curricular Affairs, clear member responsibilities	‘It is a platform for communication between students, faculty and the administration. Allows for formal and respectful communication’
			‘The SCB enhances the student experience by allowing real-time feedback to address classroom issues and improve the curriculum. Offers a mechanism for accountability and assessment’
	*Reactivity to specific issues*	Open-forum events (town hall meetings, ‘Dialogue with the Deans’), real-time communication with faculty, use of social media, creation of the special projects branch to address macro-curricular issues as they arise	‘We now receive grade reports after every test that help us understand how we compare to our peers and what objectives/items we are struggling with’
			‘More conversations about social disparities in health and more representation of the topics in curriculum’
	*Exposure to academic medicine*	Training workshops in academic medical education, collaboration with the Department of Medical Education faculty, exposure to mentors, opportunities for scholarly activities (presentation at conferences, publication)	‘I appreciate having the opportunity to take an active role in shaping UICOM’s curriculum. I appreciate the relationships I was able to establish with faculty and having mentors in the area of medical education’
			‘I’ve been introduced to the practical obstacles to curriculum planning and execution’
*Areas for improvement*	*Visibility*	Bi-monthly town hall meetings, quarterly student newsletter, integration with student government, use of social media, public posting of course reports, improved student website, introduction of SCB during new student orientation	‘Depending on feasibility, it could be nice to send out notes or updates regarding decisions made by the SCB to keep students not as involved with the SCB up to date on activities occurring within the SCB’
			‘Make it clear throughout the year who is on the board and how to reach out to them. Especially for each course and what changes have been made year to year’
	*Opportunities for involvement*	Expansion of SCB positions available, creation of special projects, curricular progress, and phase 2/3 branches, focus groups	‘I wonder how to get the other 160–180 students involved, in some practical and actionable manner, in the curriculum that they are obligated to participate in and tend to have strong opinions about’
			‘Allowing more opportunities throughout the year for more students to get involved, and providing more communication directly to students about what’s going on with the SCB’
	*Faster turnaround time*	Greater push for real-time feedback, pilot of immediate post-lecture surveys, frequent follow-up to SCB recommendations, solution-oriented problem-solving	‘Easy to give timely, actionable feedback to course before the end of the semester when changes would be too late’
			‘Time sensitivity regarding response to student concerns and criticisms’
	*Focus on senior students*	Creation of the phase 2/3 branch, involvement of students in phase 2/3 curricular design, increased recruitment of more senior students to SCB	‘Increase SCB involvement within the 3rd/4th year curriculum’
			‘I am excited for the change the years below me will experience. They receive most of the benefits’

Themes about areas for continued improvement included:

- *Visibility*: Students want regular updates regarding curricular changes and highlighted the importance of learning how their feedback led to change, despite acknowledging that the change may only affect future students.
- *Opportunities for involvement*: Non-involved students wanted more opportunities to become involved, especially later in medical school. Students appreciate the skills that students develop in leadership, curriculum, teaching and advocacy.
- *Faster turnaround time*: Students want feedback discussed in real time throughout a course or clerkship, not just at the very end. This allows for more rapid responses to student concerns, including day-to-day logistical issues and larger macro-curricular concerns.
- *Focus on senior students*: Students appreciated efforts to focus on clerkship and post-clerkship curriculum, but still perceived that the majority of student-led efforts focused on pre-clerkship curriculum.

## Discussion

Student engagement is a vital component of medical education, and medical students offer a unique perspective that adds value to curriculum design, implementation and evaluation. The UICOM-Chicago SCB is an innovative organisation that engages medical students in curriculum by providing structured opportunities across all years of training. Our survey of 563 students indicates that a majority view this student engagement as highly valuable.

Most medical schools involve students in some way; however, structural details and how these opportunities are perceived by students are lacking in the literature [7–9, 11]. In contrast, the organisation described here offers an innovative, formal program for training in and exposure to academic medicine, by engaging learners as key stakeholders in the medical education community. After SCB members are selected, they receive formal training from medical education faculty on how to identify causes of students’ concerns, gather and interpret data systematically, and present this feedback respectfully to faculty in a way that emphasises potential solutions that suit all stakeholders. Feedback is used in multiple ways beyond just course evaluation, including faculty development, promotion and tenure. Student-faculty reports are considered formal components of the administrative review process, often reducing time that faculty spend on completing formal reports of their own courses. Students host regular town hall meetings with the student body and hold longitudinal positions that encourage growth in skills and knowledge over time. Additionally, students utilise the network that the SCB provides to initiate research projects in medical education, allowing students to hone research skills and increase student-driven scholarship. This opportunity allows SCB members to gain exposure to academic medicine, facilitating the development of physician-educators [16].

Based on our analysis, we suggest the following considerations: (1) involve motivated student leaders who collaborate with peers and faculty alike and are willing to investigate curricular issues with a solutions-based approach; (2) provide students with formal training that allows for meaningful engagement in medical education; (3) systematically integrate the curricular student group into the structure of the medical school; (4) support an institution-wide cultural shift that empowers students; and (5) disseminate information regarding improvements to students and faculty in real time.

Despite potential for reproducibility at other medical schools, our study is limited in that the SCB thus far has only been implemented at UICOM-Chicago. However, the principle of organised student involvement in medical education through the methods described can easily be adapted to fit the needs and resources of other institutions. Further, although several of our students (especially those involved in the SCB) indicated increased exposure to and a desire for careers in academic medicine (Tab. 1), we did not explore long-term outcomes pertaining to involvement in the SCB. Thus, future studies will investigate the impact of this student engagement on careers in academic medicine, as well as explore perceptions of faculty and other stakeholders about this program.

In summary, by describing the novel framework of the UICOM-Chicago SCB and evaluating its impact on the student body, we highlight how medical students can add value to medical education and provide a model of best practices in student engagement in medical curriculum design, implementation and evaluation. In doing so, we provide a framework for student involvement at other institutions.
